# Composite Gels Containing Whey Protein Fibrils and Bacterial Cellulose Microfibrils

**DOI:** 10.1111/1750-3841.14509

**Published:** 2019-04-30

**Authors:** Jinfeng Peng, Vincenzo Calabrese, Julia Geurtz, Krassimir P. Velikov, Paul Venema, Erik van der Linden

**Affiliations:** ^1^ Physics and Physical Chemistry of Foods, Dept. of Agrotechnology and Food Sciences Wageningen Univ. P.O. Box 17, 6700 AA Wageningen The Netherlands; ^2^ Unilever R&D Vlaardingen, Olivier van Noortlaan 120, 3133 AT Vlaardingen The Netherlands; ^3^ Inst. of Physics, Univ. of Amsterdam, Science Park 904 1098 XH Amsterdam The Netherlands; ^4^ Soft Condensed Matter Debye Institute for NanoMaterials Science, Utrecht Univ. Princetonplein 5 3584 CC Utrecht The Netherlands

**Keywords:** bacterial cellulose microfibrils, fibrillar gel, microstructure, protein fibrils, whey protein isolate

## Abstract

In this study, we investigated the gelation of WPI fibrils in the presence of bacterial cellulose (BC) microfibrils at pH 2 upon prolonged heating. Rheology and microstructure were investigated as a function of BC microfibril concentration. The presence of BC microfibrils did not influence the gelation dynamics and resulting overall structure of the WPI fibrillar gel. The storage modulus and loss modulus of the mixed WPI‐BC microfibril gels increased with increasing BC microfibril concentration, whereas the ratio between loss modulus and storage modulus remained constant. The WPI fibrils and BC microfibrils independently form two coexisting gel networks. Interestingly, near to the BC microfibrils more aligned WPI fibrils seemed to be formed, with individual WPI fibrils clearly distinguishable. The level of alignment of the WPI fibrils seemed to be dependent on the distance between BC microfibrils and WPI fibrils. This also is in line with our observation that with more BC microfibrils present, WPI fibrils are more aligned than in a WPI fibrillar gel without BC microfibrils. The large deformation response of the gels at different BC microfibril concentration and NaCl concentration is mainly influenced by the concentration of NaCl, which affects the WPI fibrillar gel structures, changing form linear fibrillar to a particulate gel. The WPI fibrillar gel yields the dominant contribution to the gel strength.

## Introduction

The ability of proteins to form fibrils under certain conditions has been suggested as a generic feature to all proteins (Chiti & Dobson, 2006; Dobson, 2003). The subject has received considerable attention in the past decades (Adamcik & Mezzenga, 2012; Akkermans et al., 2008; Arnaudov & de Vries, 2006; Aymard, Nicolai, Durand, & Clark, 1999; Bolisetty, Harnau, Jung, & Mezzenga, 2012; Dave et al., 2013; Jung & Mezzenga, 2009; Loveday, Wang, Rao, Anema, & Singh, 2011; Nicolai, Britten, & Schmitt, 2011; Oboroceanu et al., 2011; Shimanovich et al., 2015; van der Linden, 2012). Whey protein is a class of proteins frequently studied for fibril formation (Kroes‐Nijboer, Venema, & van der Linden, 2012). A commercial system used for this purpose is whey protein isolate (WPI), containing a mixture of various types of whey protein. It is mainly composed of beta‐lactoglobulin (β‐lg), alpha‐lactalbumin (α‐lac), and bovine serum albumin (BSA) (De Wit, 1998). Upon heating WPI at 80 °C at pH 2 and low ionic strength for several hours, β‐lg was found to be the protein involved in fibril formation (Bolder, Hendrickx, Sagis, & Van der Linden, 2006a, 2006b; Bolder, Vasbinder, Sagis, & van der Linden, 2007). In fact, the protein is first hydrolyzed, due to the low pH, to peptides and part of the peptides assembles into fibrils (Akkermans et al., 2008). Above a certain WPI concentration (∼6 wt%), transparent gels consisting of these fibrils can be obtained (Bolder et al., 2006b). The fibrillar gels prepared from whey proteins by prolonged heating at pH 2 have been studied previously with respect to their gel structure and rheological properties (Aymard et al., 1999; Bolder et al., 2006b; Gosal, Clark, & Ross‐Murphy, 2004a, 2004b; Ikeda & Morris, 2002; Kavanagh, Clark, & Ross‐Murphy, 2000a, 2000b; Langton & Hermansson, 1992; Loveday et al., 2010; Loveday, Rao, Creamer, & Singh, 2009; Sagis et al., 2002). However, little research has been performed on the formation and properties of fibrillar whey protein gels mixed with other fibrillar structures.

One of such fibrillar structures is bacterial cellulose (BC) microfibrils. This has recently received considerable interest in foods as a functional material due to its multifunctionality such as gelling, thickening, stabilizing, and water binding abilities (Okiyama, Motoki, & Yamanaka, 1992, 1993; Ougiya, Watanabe, Morinaga, & Yoshinaga, 1997; Paximada, Koutinas, Scholten, & Mandala, 2016; Paximada, Tsouko, Kopsahelis, Koutinas, & Mandala, 2016; Shi, Zhang, Phillips, & Yang, 2014; Zhu et al., 2010). Their health benefits include use as a dietary fiber and low‐calorie food ingredient (i.e., fat substitute). This forms an added‐value for use in foods (Cho & Almeida, 2012; Lin & Lin, 2004; Lin, Chen, & Chen, 2011). In addition, the BC microfibrils is highly pure in comparison to structures obtained from plant cellulose (Jonas & Farah, 1998). The microfibrils of BC are ribbon‐shaped due to the interfibril hydrogen bonding and van der Waals attraction. These microfibrils have the tendency to agglomerate into a space‐filling network and exhibit gel‐like properties (Kuijk et al., 2013). The length of the microfibrils is of the order of micrometer, whereas their width is of the order of nanometer (Geyer et al., 1994). The research on BC microfibrils has mainly focused on its formation and production (Jozala et al., 2016; Tokoh, Takabe, & Fujita, 2002). Limited research has been performed to understand the interaction of BC microfibrils in food systems (Lin & Lin, 2004; Lin et al., 2011; Okiyama et al., 1992, 1993; Paximada, Koutinas, et al., 2016).

Recently, BC microfibril dispersions prepared from the well‐known dessert in Philippines, named Nata de Coco (Phisalaphong & Chiaoprakobkij, 2012), through high energy deagglomeration has been characterized by Kuijk et al. (2013) and further modified with addition of a charged polymer by Veen et al. (Veen, Kuijk, Versluis, Husken, & Velikov, 2014; Veen, Versluis, Kuijk, & Velikov, 2015). In the current study, we investigate mixed gels consisting of WPI fibrils and BC microfibrils as obtained from prolonged heating at pH 2 of a WPI solution mixed with BC microfibrils. The addition of polysaccharide to WPI has shown extensively in literature to be able to alter the gelation of WPI and thus extend its applications in foods. The aim of adding BC to WPI is to investigate whether BC can modify the WPI fibrillar structure during prolonged heating, thus, modify the properties of this WPI fibrillar gels and extend the application of this WPI fibrillar gel. Our hypothesis was that WPI still forms fibrils in the presence of BC microfibrils under this condition, and that a WPI fibrillar gel is formed in coexistence with a microfibrillar BC gel. We characterized the rheological properties, microstructure and mechanical properties of the bifibrillar gels.

## Materials and Methods

### WPI solution

WPI (Bipro, Davisco, lot # JE 198 ‐1‐420, MN, USA) powder (containing 97.5 wt% proteins) was first dissolved in an HCl solution (Merck, Germany) of pH 2 with stirring at 4 °C overnight. The concentration of the WPI in the solution is about 15 wt%. The concentration of WPI used in this study is the concentration of the WPI powder used for sample preparation. After dissolution, the pH of the WPI solution was adjusted to pH 2 with a 6 M HCl solution. We added the HCl drop by drop and made sure each drop of HCl is sufficiently mixed by stirring with a magnetic stirrer and the pH did not change further before we added the next drop. Subsequently, the solution was filtered using a syringe filter (Hydrophilic PES 0.45 µm, Millipore Millex‐HP, Germany) to remove undissolved protein. UV spectrophotometer (Cary 50 Bio, Varian, USA) and a calibration curve of known WPI concentration at a wavelength of 278 nm was used to determine the protein concentration in the solution (Kroes‐Nijboer et al., 2012). We prepared a concentration series of WPI solutions from a stock WPI solution with known concentration and measured the absorbance of each concentration, the calibration curve was constructed from the results. The WPI concentration of the final stock solution was determined by diluting to concentrations low enough to allow for UV absorbance measurement. The WPI stock solution was left at 4 °C before further sample preparation.

### BC microfibril dispersion

The dispersion of 1 wt% BC microfbrils in water was obtained from Unilever R&D Vlaardingen, The Netherlands. The detailed preparation procedure can be found in literature (Kuijk et al., 2013; Veen et al., 2014).

### WPI‐BC bifibrillar gels

We used the WPI stock solution and BC microfibril dispersion prepared above for mixture preparation. An HCl solution of pH 2 was used for dilution to reach the desired concentration ratios of WPI and BC microfibrils. The final WPI concentration in the mixtures was fixed at 9 wt% and the BC microfibril concentrations were 0, 0.05, 0.1, 0.2, 0.3, and 0.4 wt%, respectively. For homogeneous mixing, all samples were stirred for 30 min prior to analysis. The pH of the final mixture was adjusted to pH 2 using a 6 M HCl solution. For gel preparation, the mixtures were heated in a water bath (Haake Phoenix II C25P, Haake, Germany) from 20 to 80 °C with a heating rate of 2 °C/min, holding at 80 °C for 10 hr and cooling from 80 to 20 °C with a cooling rate of 2 °C/min. After heating, the mixtures were left at 20 °C for further analysis.

### Differential scanning calorimetry (DSC)

DSC was performed by placing about 50 mg sample in a sealed stainless‐steel pan in the equipment (Diamond series DSC, Perkin Elmer, Pyris, MA, USA). The measurement was performed by first equilibrating at 20 °C for 2 min; afterwards, the sample was heated from 20 to 100 °C at a heating rate of 10 °C/min, held at 100 °C for 1 min, and then cooled from 100 to 20 °C at a cooling rate of 10 °C/min. After equilibrating at 20 °C for 2 min, the sample was again heated from 20 to 100 °C at a heating rate of 10 °C/min. All samples were measured in duplicate. The software of Pyris (Perkin Elmer) was used for data analysis.

### Small deformation rheology

The WPI‐BC microfibril mixtures were stirred at room temperature for 1 hr prior to measurement. Then, the samples were transferred using a pipette with a cut‐off tip to a stress‐controlled rheometer (MCR 302, Anton Paar, Germany). Measurement was performed using sandblasted concentric cylinder geometry (CC17/TI/S‐SN38492). To prevent evaporation, the sample was covered with a thin layer of paraffin oil and a solvent trap was also used. The development of storage modulus G′ and loss modulus G′′ during gel formation was monitored at a frequency of 1 Hz and strain of 0.1%. The temperature during the gel formation was first increased from 20 to 80 °C at a heating rate of 2 °C/min, held at 80 °C for 10 hr, decreased from 80 to 20 °C at a cooling rate of 2 °C/min, and then held at 20 °C for 30 min. Afterwards, a frequency sweep was determined at a strain of 0.1% and frequency increasing from 0.01 to 100 Hz in 30 min at 20 °C. Additionally, a strain sweep was also performed at a frequency of 1 Hz and strain increasing from 0.01 to 1,000% in 30 min at 20 °C.

### Confocal laser scanning microscopy (CLSM)

One milliliter of sample is transferred to an Eppendorf. Five microliter of 0.2% Rhodamine B and 10 µL of 0.05% Calcofluor white were added to stain the WPI and BC microfibrils, respectively. The sample was vortexed for 10 s for homogeneous mixing. Sample with a volume of 125 µL was transferred into CLSM cuvettes (Gene Frame 125 µL, Thermo Scientific, Germany). The cuvettes were sealed by gently gluing a cover glass (Menzel‐glaser, Thermo Scientific) on the top. The cuvettes were then transferred and heated in a water bath (Haake Phoenix II C25P, Haake, Germany.) for gel preparation. The temperature was increased from 20 to 80 °C at a heating rate of 2 °C, held at 80 °C for 10 hr and then decreased from 80 to 20 °C at a cooling rate of 2 °C. Samples were kept at room temperature for one day before imaging. CLSM image analysis was performed on a Zeiss LSM 510 META microscope (Zeiss, Germany) equipped with an Axiovert 200 M inverted microscope using an oil immersion objective (Plan‐Apochromat 63x/1.4 oil DIC). The excitation wavelength of Rhodamine B and Calcofluor white are 543 nm and 405 nm. The emission wavelength of Rhodamine B and Calcofluor white are 560 and 420 nm.

### Scanning electron microscopy (SEM)

For image analysis of BC dispersion using SEM, a small droplet of the sample was put on a hollow copper rivet and rapidly frozen in liquid ethane. The rivets were placed in a cryo‐sample holder in liquid nitrogen and hereafter transferred to the cryo‐preparation system (MED 020/VCT 100, Leica, Vienna, Austria) onto the sample stage. Subsequently, the samples were fractured, freeze dried for 10 minutes and then sputter coated with a layer of 10 nm tungsten at the same environmental conditions. The samples were cryo‐shielded and transferred into the field emission scanning microscope (Magellan 400, FEI, Eindhoven, The Netherlands) onto the sample stage for further analysis.

For image analysis of the gels using SEM, we referred to the method described previously (Munialo, van der Linden, & de Jongh, 2016; Urbonaite et al., 2016). Gels were prepared in prelubricated 20 mL syringes for SEM imaging. The gels were cut into five pieces which are 1 cm in diameter and 1.3 cm in height and placed in 2.5% (v/v) glutaraldehyde solution for 8 hr for crosslinking of the proteins. The excess glutaraldehyde was removed by placing the gel pieces in MilliQ water with gently rotating overnight. The water was replaced gradually by 10%, 30%, 50%, 70%, and 100% acetone. The sample was gently rotated for 1 hr for each acetone concentration. Finally, the samples were left in 100% acetone. Subsequently, the sample pieces were dried using critical point drying (CPD 300, Leica, Vienna, Austria) and then were fractured and attached onto SEM sample holders using Carbon Conductive Cement (Leit‐C, Neubauer Chemicalien, Germany). The samples were stored overnight under vacuum for removal of all the solvent from the adhesive. After sputter coating with a 10 nm thick layer of iridium (SCD 500, Leica, Vienna, Austria) the fractured surfaces were analyzed on a scanning electron microscope (Magellan 400, FEI, Eindhoven, the Netherlands).

### Large deformation rheology

The WPI‐BC gels were prepared at different NaCl concentrations in order to investigate the effect of NaCl on the large deformation rheology of the gels. A stock solution of 5.9 M NaCl (Sigma Aldrich, Sodium Chloride, lot # SZBE 1630V, Germany) solution was used to reach the final NaCl concentrations of 0, 50, 100, and 200 mM in the samples. Gels were prepared in 20 mL syringes (BD plastipak, Spain) which were pre‐lubricated using paraffin oil. Cylindrical gel samples (20 mm in height and 20 mm in diameter) were obtained using a wire cutter. The fracture properties of the gels were measured by performing a uniaxial compression test on a Texture Analyser (TA‐XT plus, Stable Micro Systems, Godalming, U.K.) fitted with a 50 kg load cell and a cylindrical plastic probe of 75 mm in diameter. The gels were compressed to 80% deformation at a rate of 1 mm/s at room temperature. Four measurements were performed for each sample. The true stress, true strain and Young's modulus were calculated on the basis of the method described elsewhere (Urbonaite, de Jongh, van der Linden, & Pouvreau, 2014).

## Results and Discussion

### Macroscopic stability

**Figure 1:**
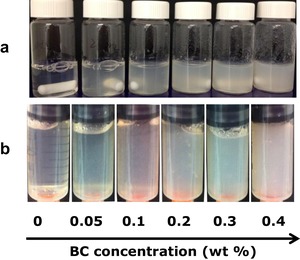
Images of WPI‐BC microfibril mixtures as a function of BC microfibril concentration at pH 2 (a) and the corresponding heat‐induced gels (b). All Samples contain 9 wt% WPI and BC microfibril concentrations range from 0, 0.05, 0.1, 0.2, and 0.3 to 0.4 wt%, as indicated below the image.

Pure WPI solution is transparent at pH 2. Adding BC microfibrils to the solution leads to a turbid mixture (Figure 1). The turbidity increases with increase in BC microfibril concentration due to the presence of thicker and long BC microfibril bundles (Kuijk et al., 2013). The mixtures showed macroscopically homogeneous and were stable at all BC microfibril concentrations. Bolder et al. (2006a, 2006b, 2007) reported that β‐lg is the only protein involved in fibril formation under the conditions used here. In the presence of BC microfibrils, the WPI gels formed are translucent and become turbid upon increasing BC microfibril concentration. The BC microfibrils do not gel upon prolonged heating at pH 2.

### Differential scanning calorimetry

**Figure 2:**
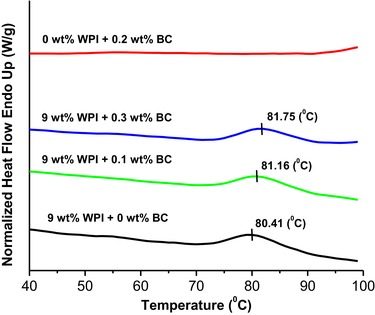
DSC thermograms for pure WPI, pure BC microfibrils and mixed WPI‐BC microfibril samples. Concentrations are indicated above the lines.

The BC microfibrils did not show endothermic peaks on heating at pH 2 (Figure 2). The WPI solution exhibited an endotherm peak upon heating due to denaturation, the peak temperature is around 80 °C. Adding BC microfibrils, to WPI solution gave a similar endothermic peak to that of the pure WPI solution, regardless of the concentrations of BC microfibrils used (Figure 2). When reheating the samples, no peaks were observed, indicating the irreversible denaturation of WPI. The thermal behavior of whey proteins has been reported to be dominated by the behavior of beta‐lactoglobulin (β‐lg). The denaturation temperature of WPI found here at low pH is in agreement with literature (Bernal & Jelen, 1985; Relkin, Eynard, & Launay, 1992), suggesting that the addition of BC in WPI solution did not influence the denaturation temperature of WPI. This is likely to denote the absence of specific interactions (Kasapis & Al‐Marhoobi, 2005) between WPI and BC microfibrils; WPI denatures in the mixtures with BC microfibrils the same way as without the BC microfibrils present.

### Rheological properties

**Figure 3:**
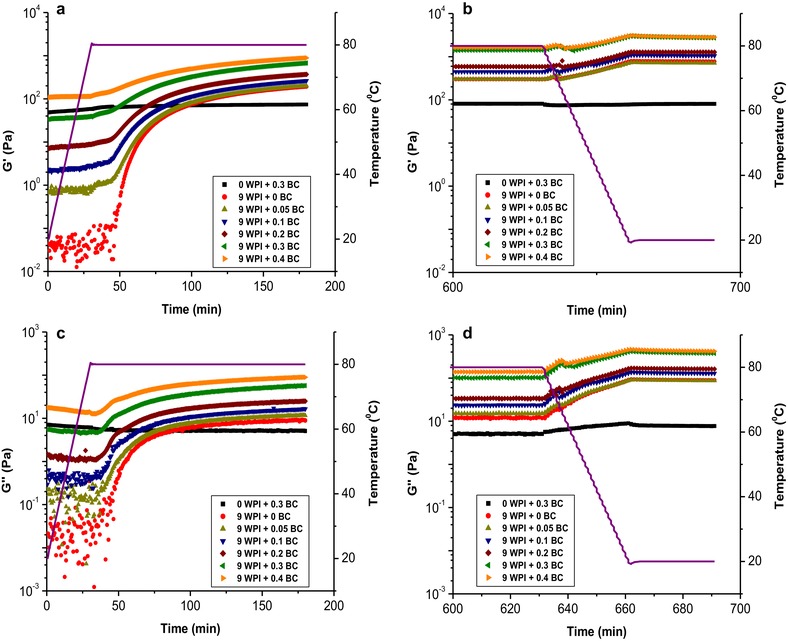
The storage modulus G′ and loss modulus G′′ development of WPI‐BC microfibril gels at pH 2 during gel formation at a strain of 0.1% and frequency of 1 Hz. (a) and (c) show the G′ and G′′ development of the samples from time 0 to 180 min. The G′ and G′′ development from time 180 to 600 min can be found in Appendix 1 of the Supporting Information. (b) and (d) show the G′ and G′′ development between the time from 600 min to 691 min. The purple line represents the temperature profile. All samples contained a fixed WPI concentration of 9 wt% and BC microfibril concentration varying from 0, 0.05, 0.1, 0.2, and 0.3 to 0.4 wt%.

For the sample containing only 0.3 wt% pure BC microfibrils (Figure 3 and Appendix 1 of the Supporting Information), the storage modulus G′ and loss modulus G′′ remained constant over time, despite the changes in temperature, indicating that the rheological properties of BC microfibrils are stable against the temperature change at pH 2. In pure WPI gels (9 wt%), the G′ and G′′ started with a small value. When temperature reached 80 °C, both G′ and G′′ underwent a sudden increase, indicating the start of WPI gelation. The G′ and G′′ reached a constant value after holding at 80 °C for 10 hr. This is in agreement with Gosal et al. (Gosal, Clark, & Ross‐Murphy, 2004b), who has studied the gelation of β‐lg at pH 2 upon prolonged heating and such sudden increase is generally recognized as a typical sol‐gel transitions of biopolymer solutions (Ross‐Murphy, 1995). Upon temperature decreasing to 20 °C, the G′ and G′′ continued to increase. A slight decrease of the G′ and G′′ was observed prior to further increase, this has been also observed in the study of Gosal et al. (2004b) and was attributed to an artefact resulted from the slippage of the gel samples after prolonged heating at an elevated temperature, which was most likely to be caused by the creep of the paraffin oil between the gel surface and the measuring cup. This creep of the paraffin oil could be caused by gel shrinkage (syneresis). At temperature of 20 °C, the G′ and G′′ values of all gels remained constant.

**Figure 4:**
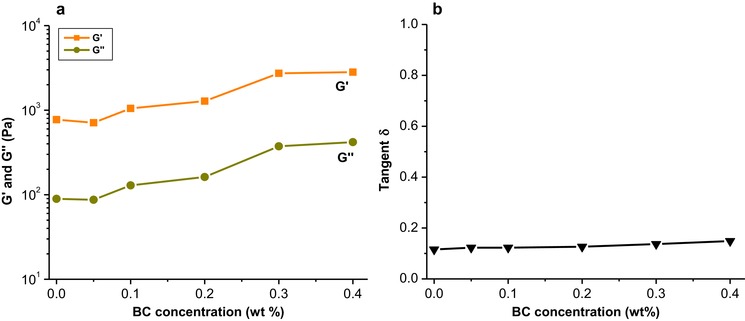
Storage modulus G′, loss modulus G′′ (a) and tangent δ (b) of WPI‐BC microfibril gels as a function of BC microfibril concentration at a fixed 9 wt% WPI. The values of G′ and G′′ are taken from the last point after holding the samples at 20 °C for 30 min. Lines are used as a guide to the eye.

Upon addition of BC microfibrils to a WPI solution, all samples have an initial G′ that is higher than G′′. The G′ values increase with increasing BC microfibril concentration. This is in line with the gel‐like structure of BC microfibrils, reported previously (Kuijk et al., 2013). We observed a behavior of the G′ and G′′ development over time similar to that of the pure WPI sample, regardless of the BC microfibril concentration. Plotting the G′ and G′′ as a function of BC microfibril concentration (Figure 4a), we observe an increase in G′ and G′′ upon increasing the BC microfibril concentration. Interestingly, the increase of G′ and G′′ as a function of BC microfibril concentration shows a similar trend, and this is further confirmed by plotting the tangent δ as a function of BC concentration (Figure 4b). Here we found that the value of tangent δ showed only a slight increase with increase of BC microfibril concentration. Although BC microfibrils are non‐gelling agents upon heating, this result implies that the rheological properties of the mixed gel are mainly dominated by WPI and that the gel structure in the WPI‐BC microfibril gel is the same as that of the pure WPI gel structure (Agoda‐Tandjawa, Durand, Gaillard, Garnier, & Doublier, 2012).

**Figure 5:**
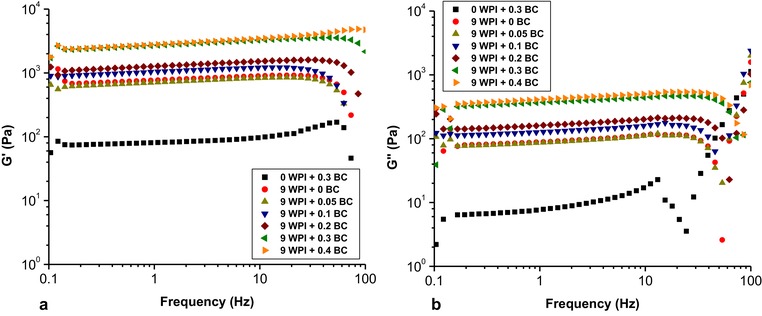
The storage modulus G′ (a) and loss modulus G′′ (b) as a function of frequency at a strain of 0.1%. All samples contain the same WPI concentration of 9 wt% and BC microfibril concentrations ranging from 0, 0.05, 0.1, 0.2, and 0.3 to 0.4 wt%.

**Figure 6:**
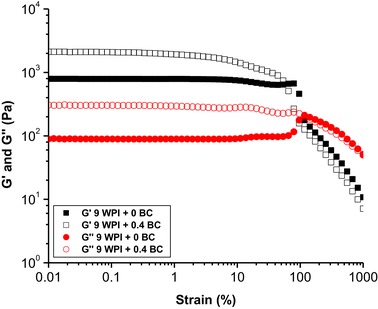
The storage modulus G′ and loss modulus G′′ as a function of strain at a frequency of 1 Hz for samples of 9 wt% pure WPI fibrillar gel and bifibrillar gel containing 9 wt% WPI and 0.4 wt% BC microfibrils.

When applying a frequency sweep (Figure 5), the G′ and G′′ of the pure BC microfibrils sample showed an independency of frequency up to ∼10 Hz. In contrast to the pure BC microfibril gel, the pure WPI gel showed an independency of frequency up to ∼30 Hz. With addition of BC microfibrils into WPI gel, the G′ and G′′ show the same trend to that of pure WPI gel upon increasing frequency, implying that the rheological properties of the WPI‐BC microfibril gels are dominated by the WPI gel structure. A strain sweep (Figure 6) was performed to confirm whether adding BC microfibrils to WPI gels affects the linear viscoelastic region (LVR). The results did not show significant change on the LVR of the gels. In addition, the results imply that the strain applied (0.1%) in the rheological tests are all in the linear regime for all samples.

Increasing the BC microfibril concentration did result in a higher storage modulus and loss modulus of the gels. In protein‐polysaccharide gels this effect was often observed and attributed to segregative phase separation (Bertrand & Turgeon, 2007; Bryant & McClements, 2000; Fitzsimons, Mulvihill, & Morris, 2008). However, according to the macroscopic stability of the WPI‐BC mixtures (Figure 1), it is unlikely that phase separation occurred. It would be more likely due to the water becoming preferably situated in the phase of BC, thereby effectively concentrating the protein phase (Beaulieu, Turgeon, & Doublier, 2001; Zasypkin, Braudo, & Tolstoguzov, 1997).

### Confocal laser scanning microscopy

**Figure 7:**
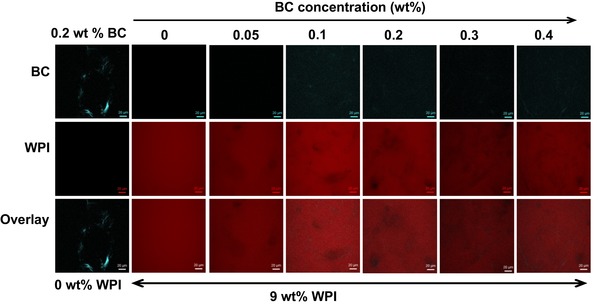
CLSM images of WPI‐BC microfibril gels containing 9 wt% WPI (shown in red) and BC microfibril (shown in green) concentrations of 0, 0.05, 0.1, 0.2, 0.3, and 0.4 wt%. On the top of the images show the BC microfibril concentrations (wt%) and at the bottom show WPI concentrations (wt%). Images from the BC microfibril channel, WPI channel, and the overlay image are shown. The scale bar corresponds to 20 µm.

To investigate the WPI‐BC microfibril gel microstructure, CLSM images were taken after gel formation. Figure 7 shows the CLSM images as a function of BC microfibril concentration. Pure BC microfibrils exhibited an inhomogeneous microstructure (Figure 7). Flocs of BC microfibril bundles are visible in the CLSM images, in line with the study of Kuijk et al. (2013), where they have shown that the BC microfibril dispersion is composed of fibril bundles, flocs, and voids, resulting in highly heterogeneous structure. Pure WPI forms a homogeneous gel at pH 2 consisting of linear fibril aggregates with sizes too small to be visible in CLSM (Aymard et al., 1999; Bolder et al., 2006b; Gosal et al., 2004a; Kavanagh et al., 2000a; Langton & Hermansson, 1992), as confirmed in Figure 7. The network of BC microfibrils must become denser with increasing BC microfibril concentrations in the mixed gel, but this cannot be concluded from CSLM images. The two gel structures do not seem to influence one another. Phase separation was not observed in the CLSM images. The signal for BC microfibrils was too weak to be clearly visualized in WPI gels, in contrast to the pure BC microfibrils sample. This could be explained by the large amount of protein particles surrounding, covering or absorbing on the surface of the BC microfibrils. We still observe the BC microfibrils present in the overlay images, showing as the darker areas. This is also confirmed by the image from the transmission channel (not shown here) of the CLSM.

### Scanning electron microscopy

SEM images were taken to obtain the details of the gel morphology. Images were taken at different magnifications and different locations within the sample. In particular, we focused on sample area where mainly WPI gel network is shown and area where both WPI and BC are present.

**Figure 8:**
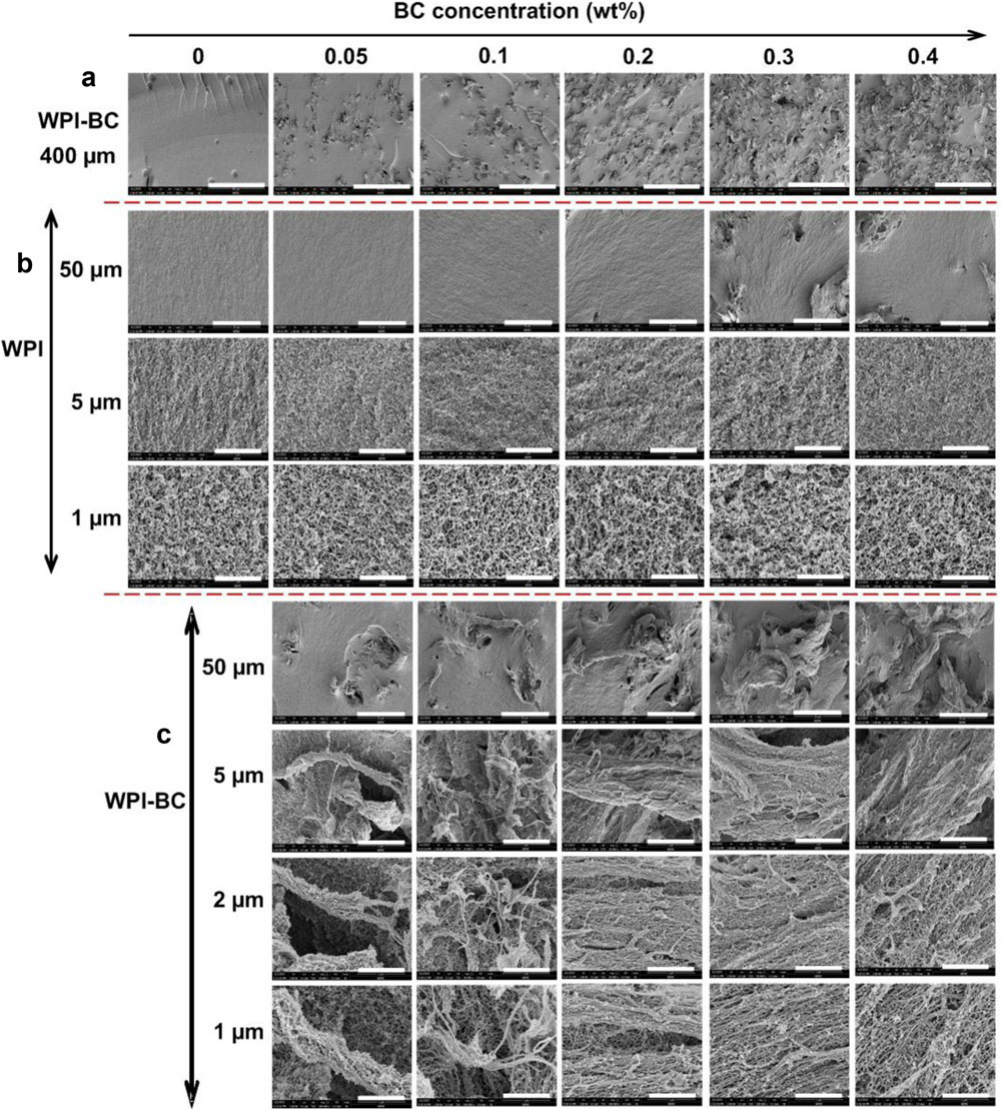
SEM images of WPI‐BC microfibril gels containing 9 wt% WPI and BC microfibril concentration of 0, 0.05, 0.1, 0.2, 0.3, and 0.4 wt%. (a) Shows the overall structure of the gels, (b) shows the structure at locations where mainly WPI is present, and (c) shows the structure at locations where both WPI and BC microfibrils are present. Scale bars correspond to 400, 50, 5, 2, and 1 µm.

From Figure 8(a), one can see that the pure WPI gels formed at pH 2 shows a smooth and homogeneous gel structure. With addition of BC microfibrils, we observed two gel structures overlapping in the sample, that is, a homogeneous WPI gel and BC microfibril gel spreading over the system without much influence on the WPI gel. The overall gel structure becomes inhomogeneous and the inhomogeneity is increased with increase in BC microfibril concentration due to the presence of BC microfibril bundles. When focused on an area where only a WPI gel network is present, like that in a pure WPI gel, several thin linear aggregates composed of peptides (Akkermans et al., 2008) are observed; corresponding to the fibril WPI based structures. When comparing the WPI gel structure at different BC microfibril concentrations to the structure of the pure WPI gel, as shown in Figure 8(b) at different magnifications, we observe that the fibrils in the WPI gel formed in the presence of BC microfibrils seemed to be better aligned, in contrast to the more random oriented fibrillar structures in a pure WPI gel. Upon increasing BC microfibril concentration, it seemed that the alignment of the WPI fibrils became more pronounced. When zooming in on the area where both WPI fibrils and BC microfibrils are present (Figure 8c), we observe the WPI fibrils seemingly attached to the BC microfibrils. The BC microfibril gel structure did not show significant changes as a function of its concentration in WPI fibrillar gels. In Appendix 2 of the Supporting Information, images taken in a different location in the sample are shown. We observed the same WPI fibril alignment along the BC microfibrils. The alignment of WPI fibrils is enhanced with increase of BC microfibril concentration in the WPI gel.

**Figure 9:**
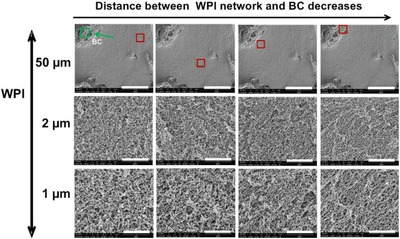
SEM images showing the WPI structure (red square) as a function of the distance to the position where BC microfibrils are present. From left to right, the distance between WPI and BC microfibrils decreases. Scale bars correspond to 50, 2, and 1 µm.

Interestingly, the extent of observed alignment of WPI fibrils varies within the sample, that is, in some areas, the WPI fibrils structure show similar to the pure WPI fibril structure, whereas in other areas, the WPI fibrils seems to better align to each other. It seems that the alignment depends on how far a specific area of WPI is from the neighboring BC microfibrils. To confirm this, the effect of the distance between WPI and BC fibrils on the WPI fibril structure is investigated. To do this, we fixed the area where BC microfibrils are present (shown in green in Figure 9) and imaged the structure of the neighboring WPI fibrils (shown in red) as a function of their distance to BC microfibrils, as shown in Figure 9. Results showed that in the area where BC microfibrils are present, the neighboring WPI fibrils seemed to be better aligned than the WPI fibrils that are farther away from the BC microfibrils.In other words, the closer the WPI is located to BC microfibrils, the better aligned the WPI fibrils are. When there are no BC microfibrils around the WPI or the BC microfibrils are far enough from the WPI, the WPI fibril structure looks similar to that of a pure WPI fibrillar gel.

The morphology of the gels from SEM reveals that adding BC microfibrils to WPI fibril forming solutions resulted upon heating in the formation of a bifibrillar gel structure. Interestingly, BC microfibrils seems to serve as a director for WPI fibril formation, leading to formation of WPI fibrils aligned next to one another when in the neighborhood of BC microfibers. Increasing the BC microfibril concentration seemingly enhanced the alignment of WPI fibrils next to one another in the neighbourhood of the BC microfibrils.

### Large deformation properties

**Figure 10:**
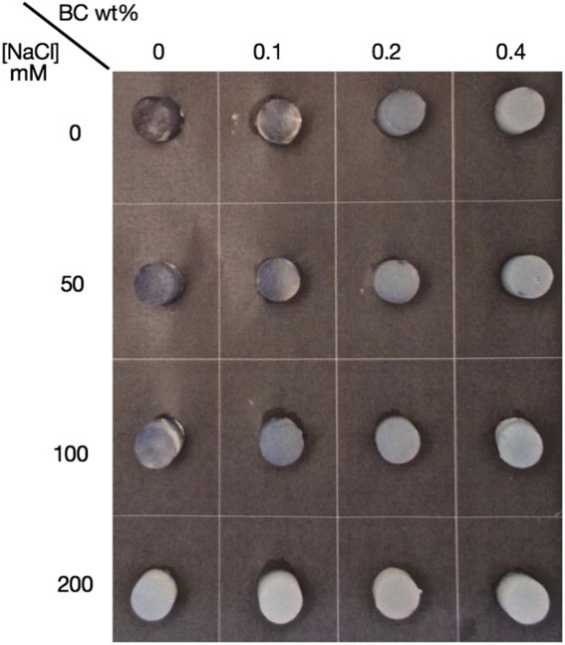
Macroscopic images of WPI‐BC microfibril gels prepared at NaCl concentration of 0, 50, 100, and 200 mM after 1 day of the preparation. All samples containing the same WPI concentration of 9 wt% and BC microfibril concentrations of 0, 0.1, 0.2, and 0.4 wt% as indicated on the top of the images. The NaCl concentrations (mM) are indicated on the left side of the images.

Large deformation rheological properties were measured to investigate the effect of BC microfibrils on the fracture properties of the WPI gel. Moreover, NaCl was also added to the mixture, the concentrations used are 0, 50, 100, and 200 mM. The dependency of WPI gel structure on ionic strength at pH 2 has been studied previously by several authors (Aymard et al., 1999; Kavanagh et al., 2000a, 2000b; Loveday et al., 2010; Sagis et al., 2002). WPI gels formed at pH 2 were found to be transparent up to a salt concentration of 100 mM (Sagis et al., 2002). With increase in salt concentration, the electrostatic repulsion is reduced and the fibrils formed become shorter and more flexible (Arnaudov & de Vries, 2006; Aymard et al., 1999; Kavanagh et al., 2000a; Loveday et al., 2010; Sagis et al., 2002). This further results in different gel structures (Arnaudov & de Vries, 2006; Loveday et al., 2010). However, structure of BC microfibrils are stable against salt concentration, as concluded from the TEM and CLSM images (results not shown). No significant difference of BC microfibril microstructure was observed. From the gel macroscopic images shown in Figure 10, the appearance of gels from pure WPI changed from transparent to more opaque with increase in salt concentration, which is in line with previous studies (Sagis et al., 2002). Increasing BC microfibril concentration resulted in more opaque gels as discussed above.

**Figure 11:**
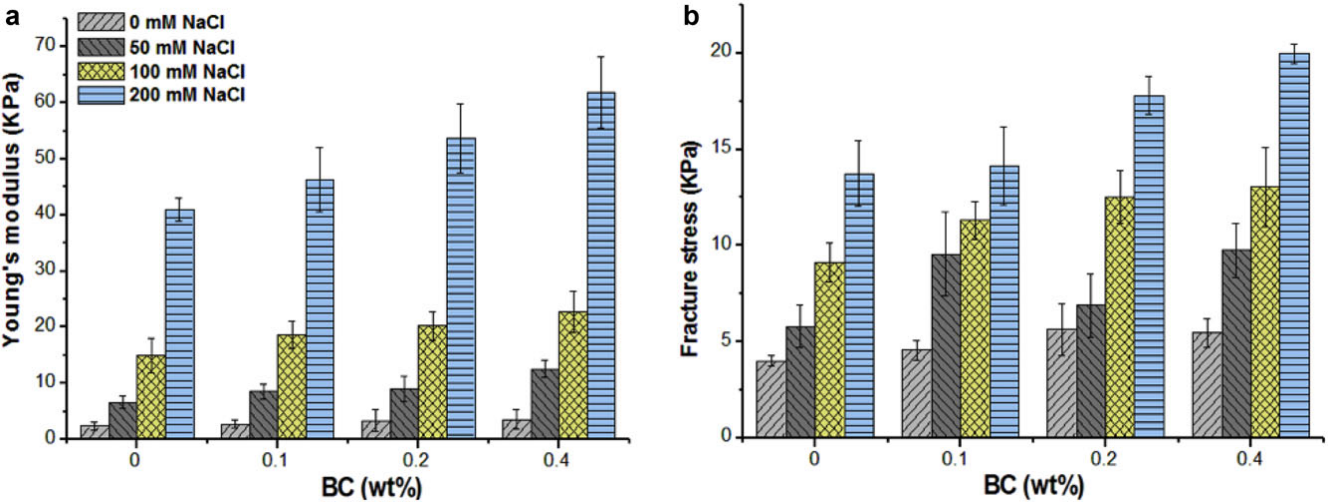
Young's modulus (a) and fracture stress (b) of WPI‐BC gels as a function of BC microfibril concentration with NaCl concentrations of 0, 50, 100, and 200 mM. All samples contain a WPI concentration of 9 wt% and BC concentrations range between 0, 0.1, 0.2, and 0.4 wt%. The error bar is based on the standard deviation of quadruplicate samples.

As shown in Appendix 3A of the Supporting Information, at 0 mM NaCl, upon addition of BC microfibrils, the WPI‐BC microfibril gels showed a similar correlation for true stress as a function of true strain to that of the pure WPI gel. All gels fractured at almost the same strain upon compression. However, the fracture stress is increased in the presence of BC microfibrils, but there is no significant difference for different BC microfibril concentrations (Figure 11b). This corresponds to small deformation properties as discussed above, where we found that addition of BC microfibrils increases the gel strength. With increasing NaCl concentration, the fracture stress of pure WPI gels increased, whereas the fracture strain decreased. Higher NaCl concentrations yield a coarser gel structure. In the mixed WPI‐BC microfibril gels, with increase in NaCl concentration, the gels fracture at lower strain and higher stress. In general, the WPI‐BC microfibril gels showed similar characteristics upon compression to that of a pure WPI gel at the same NaCl concentration. The presence of NaCl only influences the WPI gel structure, not the BC microfibril structure.

The Young's modulus reflects the stiffness of the gel. As shown in Figure 11(a), increasing NaCl concentration increases the stiffness of the pure WPI gel. Upon addition of BC microfibrils, the gels become stiffer at all NaCl concentrations upon increasing BC microfibril concentration.

Overall, the large deformation results suggest that the concentration of NaCl only influences the WPI structure with according changes in rheological behavior and fracture properties.

## Conclusion

We have prepared bi‐fibrillar gels from WPI and BC microfibrils at pH 2 upon prolonged heating. In the presence of BC microfibrils, the WPI forms a fibrillar gel consisting of linear fibrillar aggregates. The two types of fibrillar structures co‐exist and are overall homogeneous under the conditions studied. Increase of BC microfibrils increases the firmness of the gel. Upon increasing NaCl concentration in the bi‐fibrillar gel, the WPI structure changes similarly as the WPI structure without the BC microfibrils present. The WPI fibrillar structure dominates the rheological properties of the bi‐fibrillar gels. When fibrils from WPI are being formed near BC micofibrils, the BC microfibrils seemed to serve as a director for the WPI fibril formation. With the presence of BC around the WPI, the fibrils formed looked better aligned next to one another parallel to the BC microfibrils.

## Supporting information


**Appendix 1**–The storage modulus G’ (a) and loss modulus G’’ (b) development of WPI‐BC microfibril gels at pH 2 during gel formation at a strain of 0.1% and frequency of 1 Hz from time 180 to 600 min. The purple line represents the temperature profile. All samples contained a fixed WPI concentration of 9 wt% and BC microfibril concentration varying from 0, 0.05, 0.1, 0.2, and 0.3, to 0.4 wt%.
**Appendix 2**–SEM images of WPI‐BC gels containing 9 wt% WPI and BC concentration of 0, 0.05, 0.1, 0.2, 0.3, and 0.4 wt% BC. Images show the structure at locations where both WPI and BC microfibrils are present. Scale bars correspond to 50, 5, 2, and 1 µm.
**Appendix 3**–Uniaxial compression with 80% deformation of WPI‐BC gels at pH 2 with NaCl concentrations of 0 (A), 50 (B), 100 (C), and 200 (D) mM. All samples contain a WPI concentration of 9 wt% and BC microfibril concentrations range between 0, 0.1, 0.2, and 0.4 wt%.
