# Supplementary material for: Composite Gels Containing Whey Protein Fibrils and Bacterial Cellulose Microfibrils
Source: J Food Sci. 2019 Apr 30;84(5):1094–103. doi: 10.1111/1750-3841.14509 (PMC6593742; doi:10.1111/1750-3841.14509)
Supplement: Supplementary file 1 — Appendix 1–The storage modulus G’ (a) and loss modulus G’’ (b) development of WPI‐BC microfibril gels at pH 2 during gel formation at a strain of 0.1% and frequency of 1 Hz from time 180 to 600 min. The purple line represents the temperature profile. All samples contained a fixed WPI concentration of 9 wt% and BC microfibril concentration varying from 0, 0.05, 0.1, 0.2, and 0.3, to 0.4 wt%. Appendix 2–SEM images of WPI‐BC gels containing 9 wt% WPI and BC concentration of 0, 0.05, 0.1, 0.2, 0.3, and 0.4 wt% BC. Images show the structure at locations where both WPI and BC microfibrils are present. Scale bars correspond to 50, 5, 2, and 1 µm. Appendix 3–Uniaxial compression with 80% deformation of WPI‐BC gels at pH 2 with NaCl concentrations of 0 (A), 50 (B), 100 (C), and 200 (D) mM. All samples contain a WPI concentration of 9 wt% and BC microfibril concentrations range between 0, 0.1, 0.2, and 0.4 wt%. [file JFDS-84-1094-s001.docx]

**Appendix 1** The storage modulus G’ (a) and loss modulus G’’ (b) development of WPI-BC microfibril gels at pH 2 during gel formation at a strain of 0.1 % and frequency of 1 Hz from time 180 to 600 min. The purple line represents the temperature profile. All samples contained a fixed WPI concentration of 9 wt% and BC microfibril concentration varying from 0, 0.05, 0.1, 0.2, and 0.3, to 0.4 wt%.


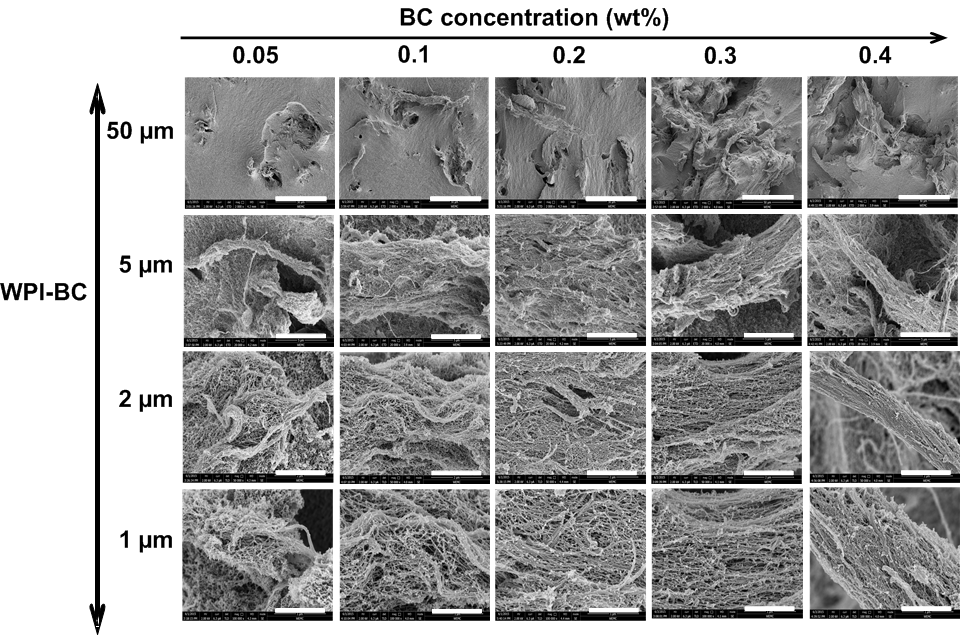


**Appendix 2** SEM images of WPI-BC gels containing 9 wt% WPI and BC concentration of 0, 0.05, 0.1, 0.2, 0.3 and 0.4 wt% BC. Images show the structure at locations where both WPI and BC microfibrils are present. Scale bars correspond to 50 µm, 5 µm, 2 µm, and 1 µm.

**Appendix 3** Uniaxial compression with 80% deformation of WPI-BC gels at pH 2 with NaCl concentrations of 0 (A), 50 (B), 100 (C), and 200 (D) mM. All samples contain a WPI concentration of 9 wt% and BC microfibril concentrations range between 0, 0.1, 0.2, and 0.4 wt%.
